# Bibliometric and visualized analysis of posterior chamber phakic intraocular lens research between 2003 and 2023

**DOI:** 10.3389/fmed.2024.1391327

**Published:** 2024-04-08

**Authors:** Jiliang Ning, Qiaosi Zhang, Wei Liang, Rui Zhang, Zequn Xing, Lin Jin, Lijun Zhang

**Affiliations:** ^1^Department of Ophthalmology, The Third People’s Hospital of Dalian, Dalian, China; ^2^Department of Ophthalmology, Dalian Municipal Eye Hospital, Dalian, China; ^3^Liaoning Provincial Key Laboratory of Cornea and Ocular Surface Diseases, Dalian, China; ^4^Liaoning Provincial Optometry Technology Engineering Research Center, Dalian, China

**Keywords:** posterior chamber phakic intraocular lens, implantable collamer lens, bibliometric analysis, VOSviewer, CiteSpace

## Abstract

**Introduction:**

Myopia is causing a major public health concern, with its prevalence increasing globally. This study aimed to discuss posterior chamber phakic intraocular lens (pIOL) research publication trends and hotspots over the past 20 years.

**Methods:**

Bibliometric analysis was performed using the Web Science Core Collection to investigate posterior-chamber pIOL research publication trends. The extracted records were analyzed, and a knowledge map was built using VOSviewer v.1.6.20. The analysis included visualizing the annual publication count, countries/regions distribution, international and institutional collaborations, author productivity, and journal contribution, in addition to identifying knowledge bases and hotspots. Burst keywords were extracted using CiteSpace v.6.1.R.

**Results:**

In total, 791 articles on posterior chamber pIOLs published between 2003 and 2023 were retrieved. China had the highest number of publications, whereas Japanese papers received the most citations. Fudan University had the highest number of publications, with articles from Kitasato University having the highest number of citations. Regarding individual research, Xingtao Zhou has published the most significant number of articles, and Shimizu Kimiya had the highest number of citations. The top productive/influential journal was ‘Journal of Cataract & Refractive Surgery’. The top cited references primarily focused on reporting the clinical outcomes of implantable collamer lens (ICL) for individuals with moderate to high myopia. The keywords primarily formed four clusters: posterior chamber pIOL clinical outcomes for myopic astigmatism correction, posterior chamber pIOL implantation complications, ICL size selection and postoperative vault predictions, and postoperative visual quality following posterior chamber pIOL implantation.

**Conclusion:**

This study presents the first bibliometric analysis of research trends in posterior chamber pIOL over the past two decades. We investigated the current state and emerging trends of global collaboration and research focal points in this field, offering fresh insights and guidance for researchers.

## 1 Introduction

The prevalence of myopia is increasing globally, causing a major public health concern. It is estimated that billions of individuals will be affected by myopia by 2050 (1). Refractive surgery is crucial in myopia treatment; it enhances patients’ quality of life, productivity, and overall daily performance (2). There are three main types of refractive surgery: laser refractive surgery, implantation and refractive lens exchange, and phakic intraocular lens implantation (pIOL) (3). Implantation using pIOL is reversible, unlike the other two surgeries. Posterior chamber pIOLs are positioned further away from the corneal endothelium, inflicting less harm than early anterior chamber angle-supported pIOLs and anterior chamber iris-fixated pIOLs (3). The Visian implantable collamer lens (ICL) (STAAR Surgical, Nidau, Switzerland) is the most widely used type of pIOL globally. It is safe and effective and corrects myopia, hyperopia, and astigmatism (4, 5). Over the last two decades, significant progress has been made in posterior chamber pIOL implantation research.

Bibliometric analysis enables the scientific and quantitative analysis of publications. It was first introduced by Pritchard in 1969 and was later expanded by Van Raan’s infographics in 2004 (6, 7). This method allows for citation, coauthor, and keyword co-occurrence analyses, which can create knowledge maps. These knowledge maps can be visualized using tools such as CiteSpace and VOSviewer.

This study evaluated growth in the annual distribution of publications, international and institutional collaborations, author productivity, journal contribution, and identifying knowledge bases and hotspots related to posterior chamber pIOLs research. Assessing research trends in the academic field is crucial in identifying gaps that require attention in future studies. Therefore, our study used bibliometric techniques to comprehensively assess the current developmental status and future trends in posterior chamber pIOLs.

## 2 Materials and methods

### 2.1 Data sources and search strategies

The Science Citation Index Extension database of the Online Web of Science Core Collection (WoSCC) was used as the research source. The search keywords were “Posterior chamber phakic intraocular lens” or “Implantable collamer lens.” The search time was between 2003 and 2023; the specified document types were articles. Language restrictions were not imposed. The search results were obtained as plain-text files and complete records with cited references. The search was conducted on January 31, 2024, and basic information on each article was collected, including the author, title, abstract, institution, journal, country, keywords, and references.

### 2.2 Analytical tools and methods

**Figure 1:**
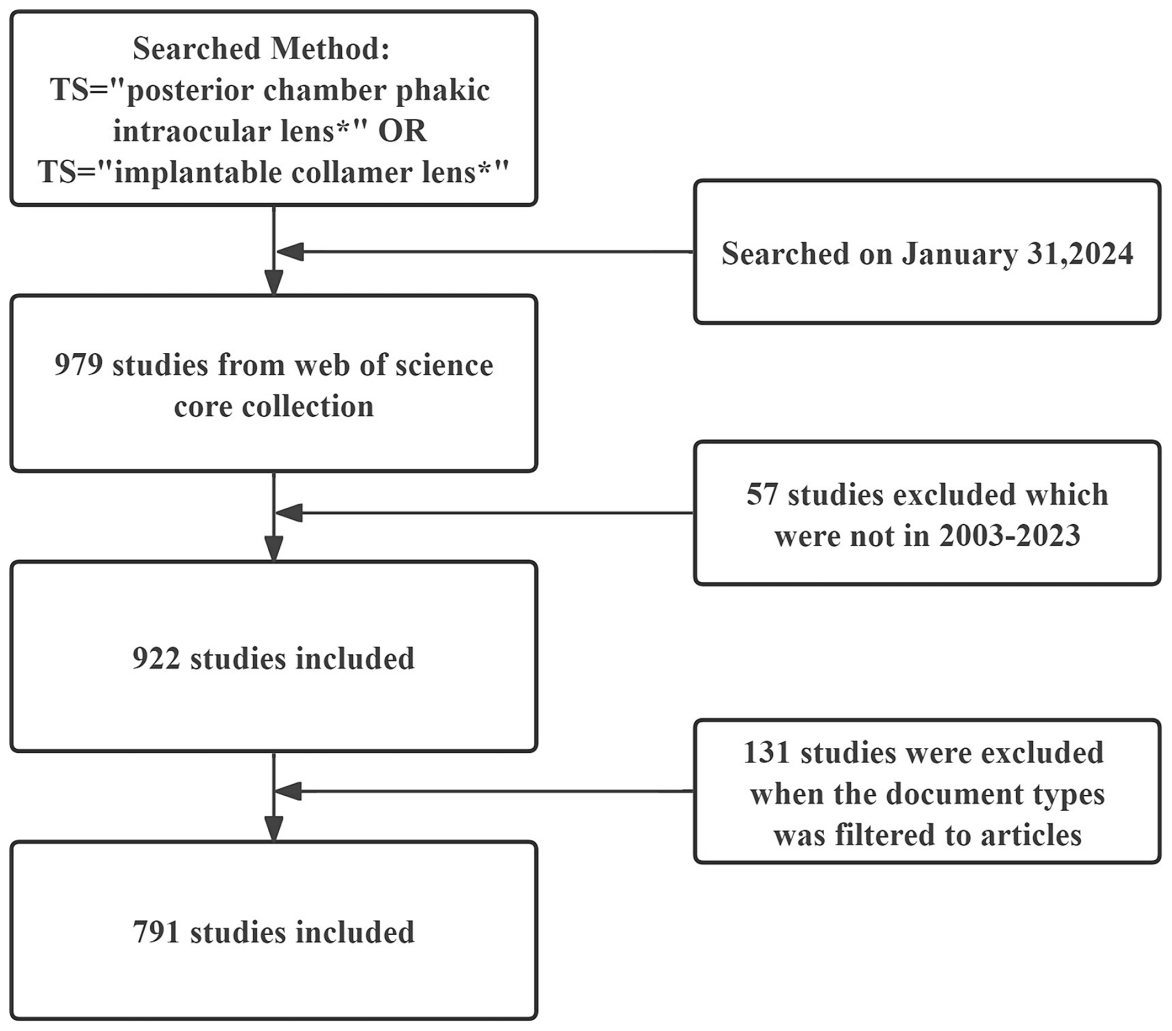
Data sources and search strategies.

Visualization software can be used to analyze the publication data and generate knowledge graphs. This study analyzed publication data, including publication year, author, country/region, research institution, journals, citations, and keywords, using VOSviewer v.1.6.20. VOSviewer,^1^ developed by van Eck and Waltman, is a literature visualization software that displays cluster analysis results (8). The knowledge graph generated by VOSviewer represents items as nodes and links. The sizes of the nodes and links correspond to the weights of the analyzed components. Node size indicates the number of publications, whereas the length and thickness of the connections between nodes represent the strength of the relationships between the analyzed components. Citation burst analysis on keywords was performed using CiteSpace 6.2.1, developed by Drexel University in Philadelphia, PA, United States. The burst map showed the burst intensity, with the red portion indicating the period during which the keywords emerged. This study utilized software to perform countries, authors, and institutional collaboration network coauthor analysis. In addition, it conducted reference co-citation analysis, co-occurrence analysis, and citation bursts of keywords (Figure 1).

## 3 Results

### 3.1 Annual quantitative distribution of literature

**Figure 2:**
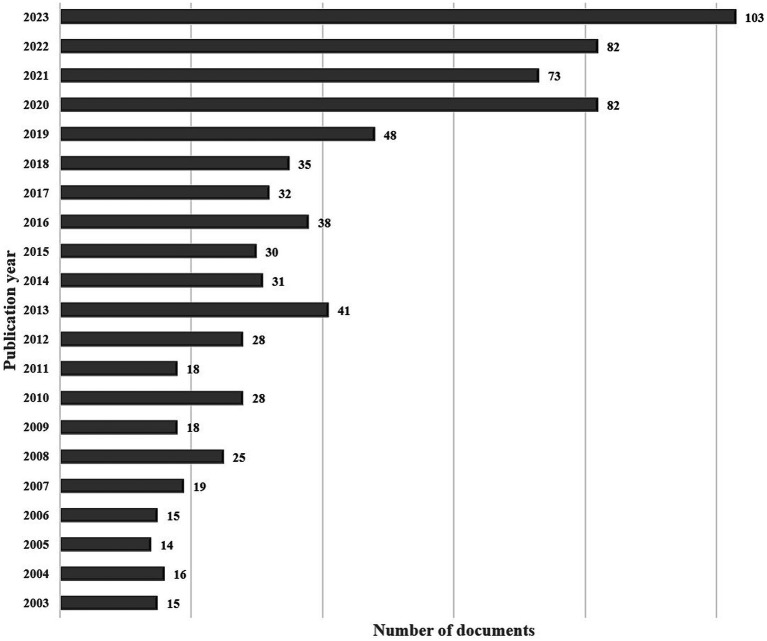
Annual number of publications in posterior chamber pIOL research between 2003 and 2023.

WoSCC indexed 791 articles published between 2003 and 2023 based on the selection criteria. The posterior chamber pIOL annual publication volume is shown in Figure 2. Over the past 20 years, posterior chamber pIOL publications have increased consistently, with a significant surge in the last four years. There were 103 publications in 2023, highlighting the rapid research development in this field.

### 3.2 Distribution and co-authorship of countries/regions

**Figure 3:**
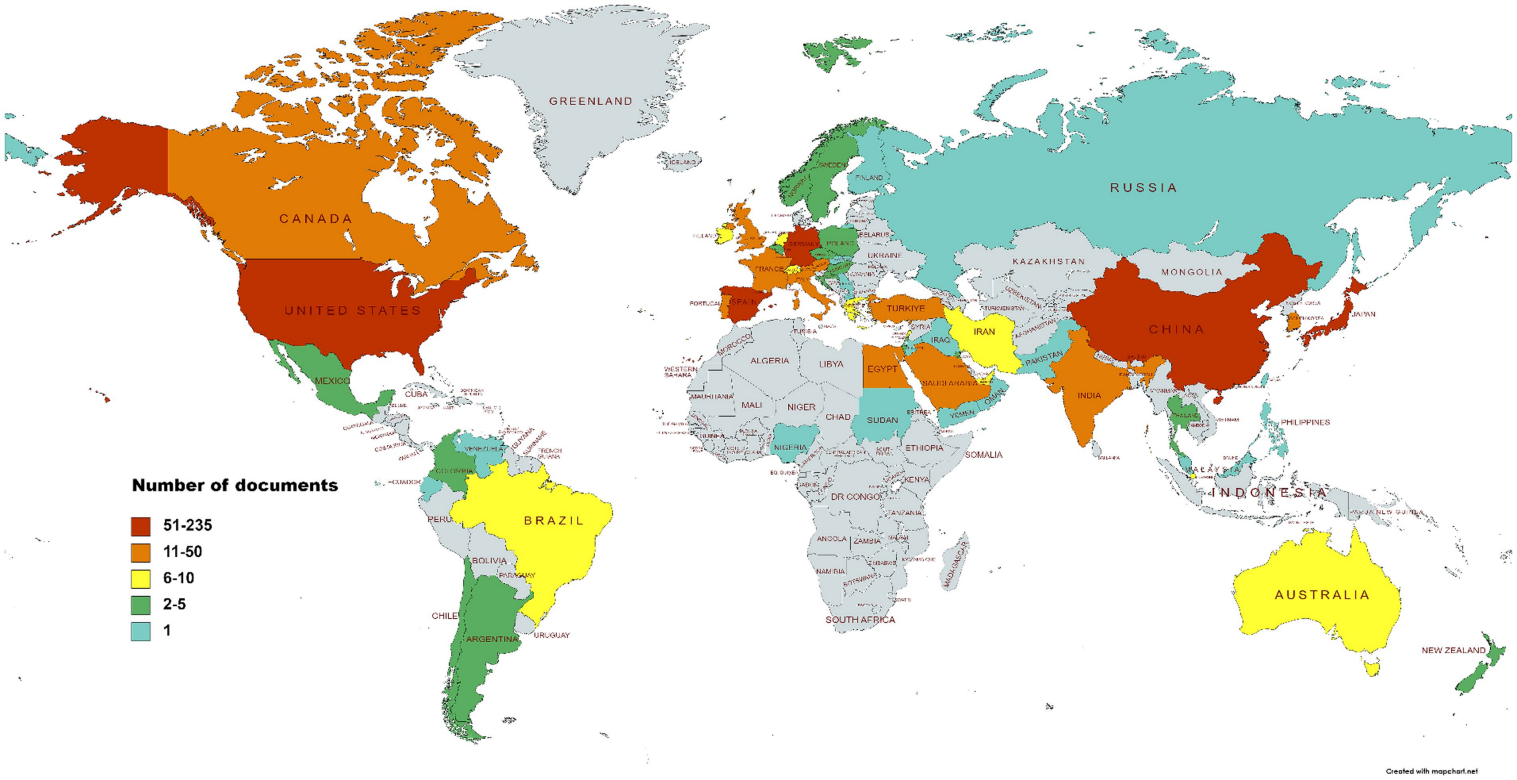
Distribution of main research countries/regions in posterior chamber pIOL research.

**Figure 4:**
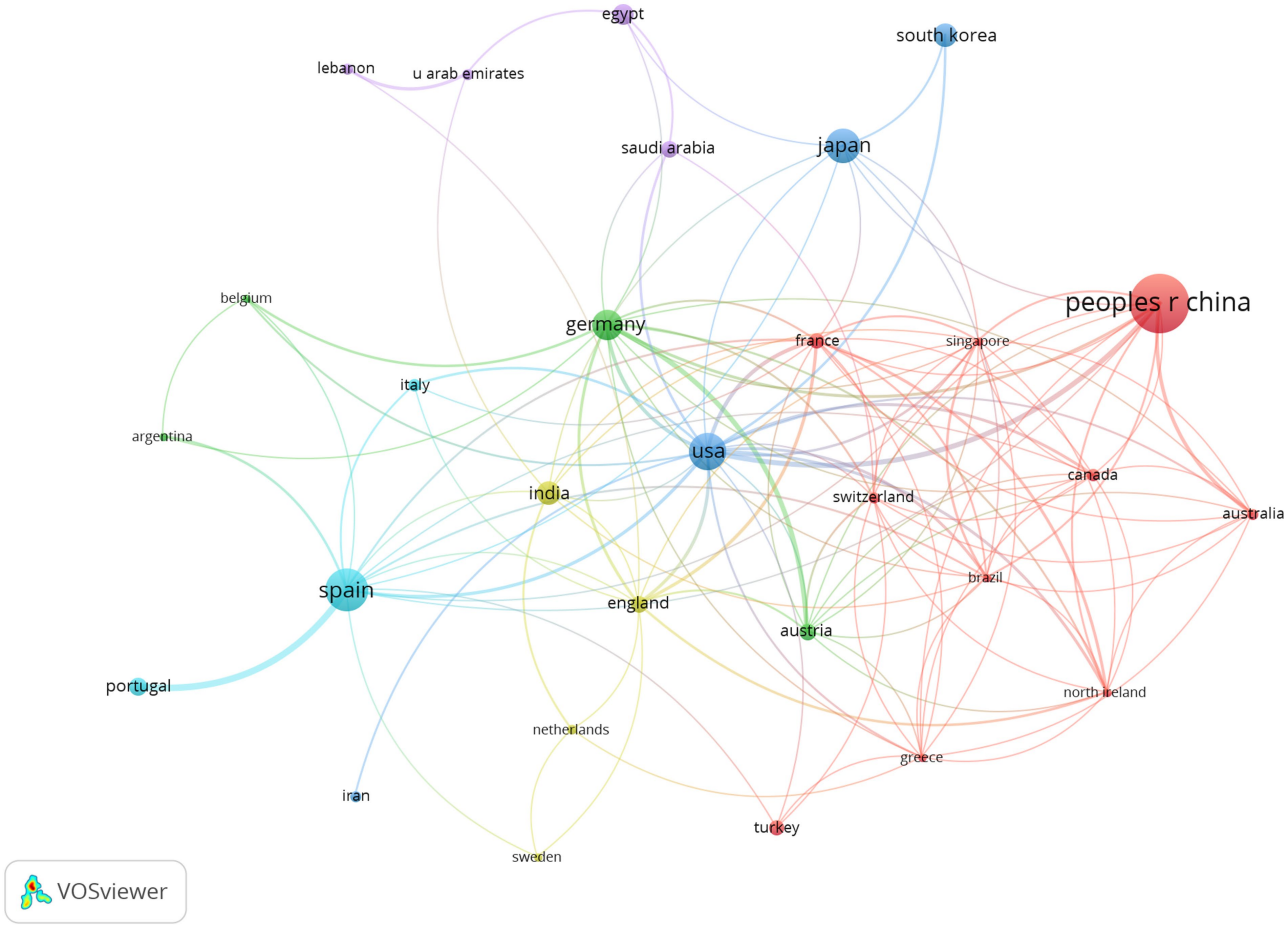
Co-authorship network of countries/regions in posterior chamber pIOL research (The minimum number of documents of a country/region was set as 5; 29 of the 62 countries involved in posterior chamber pIOL research met the threshold).

Figure 3 shows WoSCC search results of 791 articles identified from 62 countries. The top 10 countries involved in posterior chamber pIOL research published 739 articles, accounting for 93.4% of the published papers (Table 1). Regarding publication count, China produced the highest number of publications (235 publications, 29.7%), followed by Spain (123 publications, 15.5%) and the United States (92 publications, 11.6%). With respect to publication influence, Japanese publications received the highest number of citations (2,942 citations, 23%), followed by Spain (2,928 citations, 22.9%) and the United States (2,707 citations, 21.2%). A country/regional collaboration network, as illustrated in Figure 4, was created using the coauthor analysis method. The size of each node represents the number of articles published by the respective country, and the links between nodes represent collaborations. The strength of the link indicates the intensity of cooperation.

**Table 1:** Top 10 productive/influential countries/regions in posterior chamber pIOL research, 2003–2023.

Rank	Countries	Documents	Rank	Countries	Citations
1	China	235 (29.7%)	1	Japan	2,942 (23%)
2	Spain	123 (15.5%)	2	Spain	2,928 (22.9%)
3	USA	92 (11.6%)	3	USA	2,707 (21.2%)
4	Japan	81 (10.2%)	4	China	1,632 (12.8%)
5	Germany	60 (7.6%)	5	India	982 (7.7%)
6	South Korea	40 (5.1%)	6	France	915 (7.2%)
7	India	37 (4.7%)	7	South Korea	912 (7.1%)
8	Egypt	28 (3.5%)	8	Germany	686 (5.4%)
9	Portugal	24 (3.0%)	9	Brazil	637 (5%)
10	England	19 (2.4%)	10	Portugal	625 (4.9%)

### 3.3 Distribution and co-authorship of research organizations

**Figure 5:**
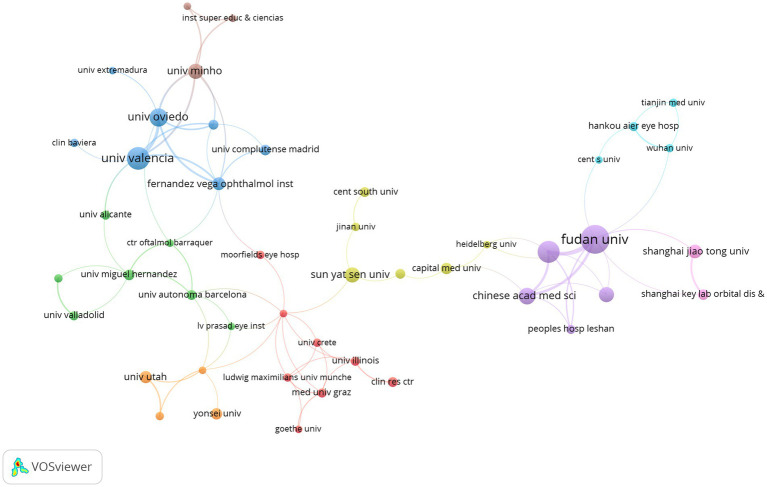
Co-authorship network institutions in posterior chamber pIOL research (The minimum number of an organization’s documents was set as 5; 66 of the 822 organizations involved in posterior chamber pIOL research met the threshold).

The 791 articles identified using WoSCC were published across 822 institutions. The top 10 institutions involved in posterior chamber pIOL research contributed 386 articles, accounting for 48.8% of the total publications (Table 2). Regarding publication count, Fudan University had the highest number of publications (68 articles, 8.6%, China), followed by Kitasato University (52 articles, 36.6%, Japan) and the University of Valencia (44 articles, 5.6%, Spain). With respect to publication impact, Kitasato University’s articles received the highest number of citations (1,657 citations, 13%, Japan), followed by the University of Valencia (1,138 citations, 8.9%, Spain) and the University of Oviedo (907 citations, 7.1%, Spain). Figure 5 shows the collaborative network of research institutions generated using coauthor analysis. The size of each node represents the number of articles published by the respective research institution, and the links between nodes indicate collaboration. The strength of these links reflects the intensity of cooperation.

**Table 2:** Top 10 productive/influential organizations in posterior chamber pIOL research, 2003–2023.

Rank	Organization (Country)	Documents	Rank	Organization (Country)	Citations
1	Fudan University (China)	68 (8.6%)	1	Kitasato University (Japan)	1,657 (13%)
2	Kitasato University (Japan)	52 (6.6%)	2	University of Valencia (Spain)	1,138 (8.9%)
3	University of Valencia (Spain)	44 (5.6%)	3	University of Oviedo (Spain)	907 (7.1%)
4	Sanno Hospital (Japan)	29 (3.7%)	4	Sanno Hospital (Japan)	825 (6.5%)
5	University of Oviedo (Spain)	29 (3.7%)	5	Center for Clinical Research (USA)	724 (5.7%)
6	Nagoya Eye Clinic (Japan)	23 (2.9%)	6	Autonomous University of Barcelona (Spain)	688 (5.4%)
7	University of Minho (Spain)	20 (2.5%)	7	University of Arizona (USA)	629 (4.9%)
8	Zhejiang University (China)	20 (2.5%)	8	L. V. Prasad Eye Institute (India)	620 (4.9%)
9	Sun Yat-sen University (China)	19 (2.4%)	9	Singapore National Eye Centre (Singapore)	618 (4.8%)
10	Keio University (Japan)	17 (2.1%)	10	University of Minho (Spain)	617 (4.8%)

### 3.4 Distribution and co-authorship of authors

**Figure 6:**
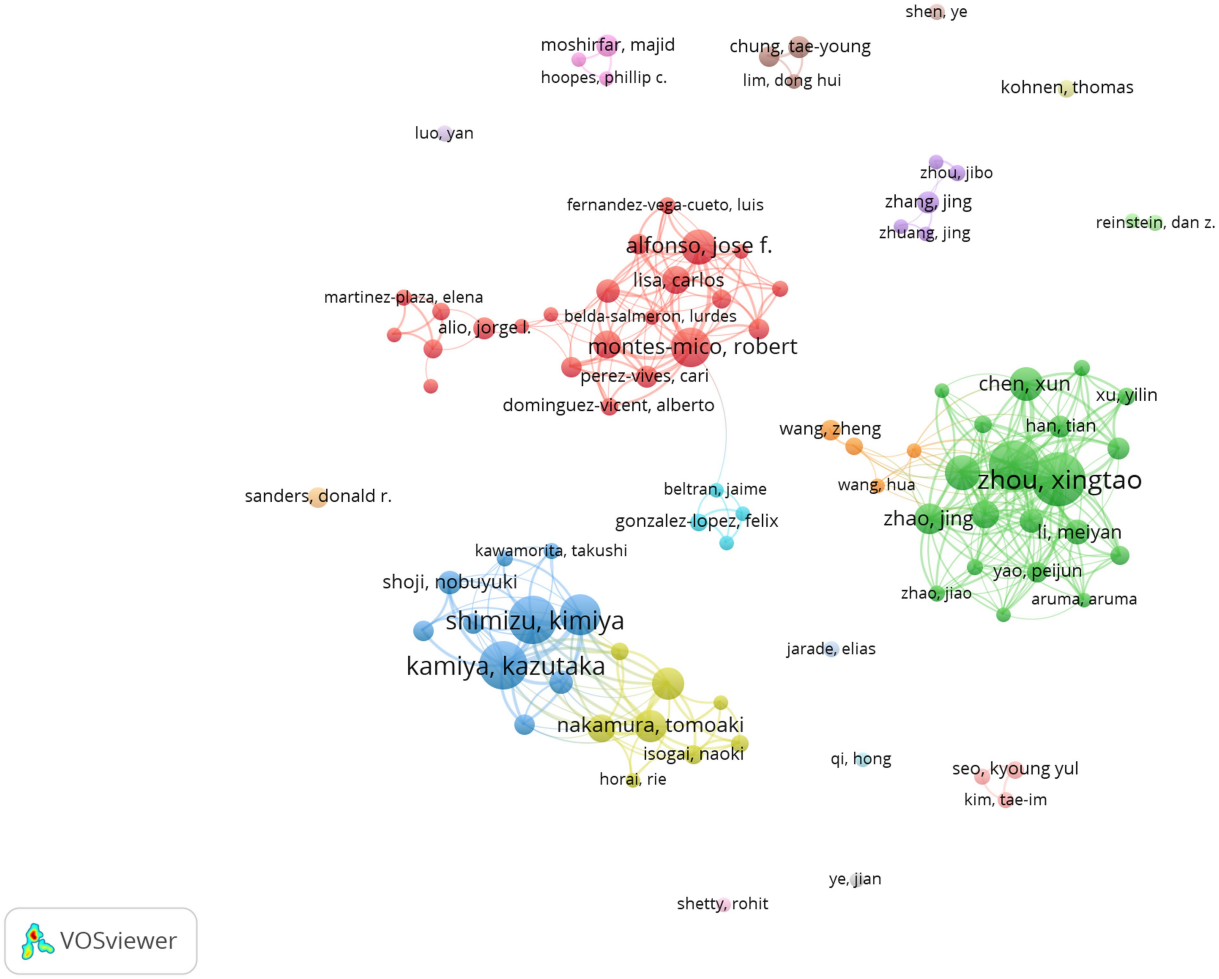
Co-authorship network of authors in posterior chamber pIOL research (The minimum number of documents of an author was set as 5; 93 of the 2,518 authors involved in posterior chamber pIOL research met the threshold).

According to the search results of WoSCC, 2,581 authors participated in posterior chamber pIOL research. Table 3 presents the top 10 authors with the highest productivity and influence in this field. Regarding productivity, Xingtao Zhou from China published the highest number of articles (61 articles), followed by Xiaoying Wang (52 articles, China) and Kamiya Kazutaka (50 articles, Japan). Regarding influence, Shimizu Kimiya from Japan has the highest number of citations (1,742 citations), followed by Kamiya Kazutaka (1,648 citations, Japan) and Igarashi Akihito (1,361 citations, Japan). Figure 6 shows the authors’ collaboration network generated using the coauthor analysis. The size of each node represents the number of articles published by the respective research institutions, and the links between the nodes represent collaborations. The strength of the link indicates the intensity of cooperation.

**Table 3:** Top 10 productive/influential authors in posterior chamber pIOL research, 2003–2023.

Rank	Author(Countries)	Documents	Rank	Author(Countries)	Citations
1	Zhou XT (China)	61	1	Shimizu K (Japan)	1742
2	Wang XY (China)	52	2	Kamiya K (Japan)	1,648
3	Kamiya K (Japan)	50	3	Igarashi A (Japan)	1,361
4	Shimizu K (Japan)	48	4	Montes-Mico R (Spain)	950
5	Igarashi A (Japan)	35	5	Alfonso JF (Spain)	853
6	Montes-Mico R (Spain)	33	6	Nakamura T (Japan)	582
7	Alfonso JF (Spain)	27	7	Komatsu M (Japan)	581
8	Niu LL (China)	26	8	Zhou XT (China)	569
9	Chen X (China)	25	9	Ambrósio R (Brazil)	567
10	Nakamura T (Japan)	23	10	Belin MW (USA)	567

### 3.5 Contribution and citation analysis of journals

**Figure 7:**
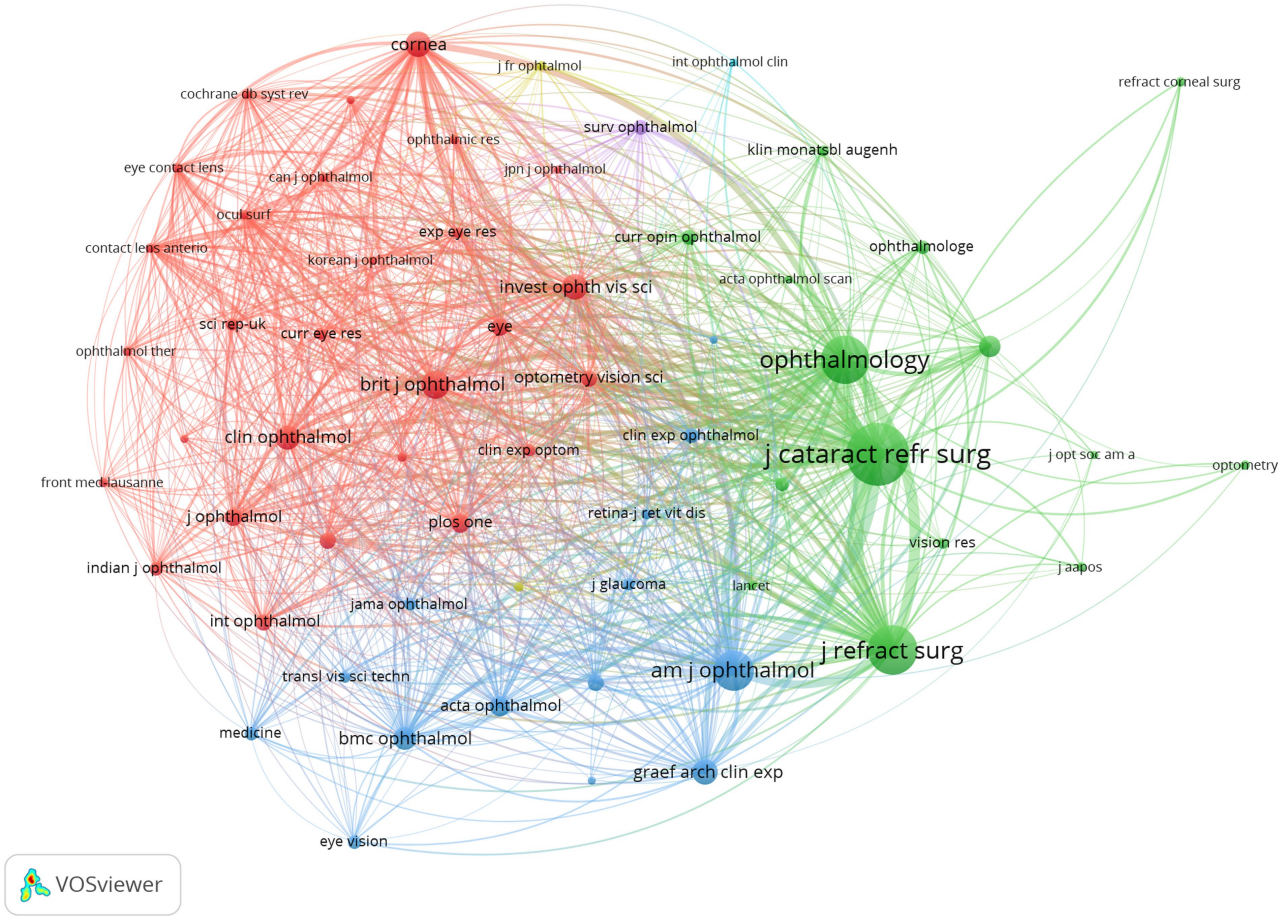
Citation network of journals in posterior chamber pIOL research (The minimum number of documents of a journal was set as 5; 31 of the 100 journals involved in posterior chamber pIOL research met the threshold).

The analysis of 791 articles using WoSCC revealed that they were published across 100 journals. Table 4 displays the Top 10 productive/influential journals in posterior chamber pIOL research. The top three journals in terms of productivity are ‘Journal of Cataract & Refractory Surgery’, ‘Journal of Refractive Surgery’, and ‘BMC Ophthalmology’, with 118, 98, and 55 articles, respectively. ‘Journal of Cataract & Refractive Surgery’ stands out with 2,701 citations, establishing it as the most influential journal in the field. Figure 7 illustrates the citation network of the journal through citation analysis. The size of each node corresponds to the number of articles published by the research journals, while the links between nodes signify collaborations. The strength of the link reflects the level of cooperation.

**Table 4:** Top 10 productive/influential journals in posterior chamber pIOL research, 2003–2023.

Rank	Journal	Documents	Rank	Journal	Citations
1	Journal of Cataract & Refractive Surgery	118	1	Journal of Cataract & Refractive Surgery	2,701
2	Journal of Refractive Surgery	98	2	Journal of Refractive Surgery	1814
3	BMC Ophthalmology	55	3	Ophthalmology	1,419
4	American Journal of Ophthalmology	41	4	American Journal of Ophthalmology	1,375
5	Graefe’s Archive for Clinical and Experimental Ophthalmology	28	5	Cornea	757
6	International Journal of Ophthalmology	26	6	Graefe’s Archive for Clinical and Experimental Ophthalmology	478
7	International Ophthalmology	24	7	British Journal of Ophthalmology	425
8	European Journal of Ophthalmology	23	8	BMC Ophthalmology	401
9	Ophthalmology	22	9	Acta Ophthalmologica	251
10	Clinical Ophthalmology	21	10	PLOS One	218

### 3.6 Co-citation analysis of reference

In total, 8,119 references were cited in 791 publications. Notably, 166 documents met the threshold when the minimum number of citations for cited documents was set at 20. Table 5 lists the top 10 cited documents. The most cited reference is ‘United States Food and Drug Administration clinical trial of the Implantable Collamer Lens (ICL) for moderate to high myopia: three-year follow-up’ published in Ophthalmology in 2004.

**Table 5:** Top 10 cited references in posterior chamber pIOL research, 2003–2023.

Rank	Title	Citations	Year	Author
1	United States Food and Drug Administration clinical trial of the Implantable Collamer Lens (ICL) for moderate to high myopia: three-year follow-up (PMID: 15350323)	223	2004	Sanders DR
2	US food and drug administration clinical trial of the implantable contact lens for moderate to high myopia (PMID:12578765)	151	2003	Vukich JA
3	Implantable contact lens for moderate to high myopia: relationship of vaulting to cataract formation (PMID:12781276)	134	2003	Gonvers M
4	Implantable collamer posterior chamber intraocular lenses: a review of potential complications (PMID: 21710954)	121	2011	Fernandes P
5	Eight-year follow-up of posterior chamber phakic intraocular lens implantation for moderate to high myopia (PMID: 24239774)	119	2014	Igarashi A
6	Meta-analysis and review: effectiveness, safety, and central port design of the intraocular collamer lens (PMID: 27354760)	118	2016	Packer M
7	Safety of posterior chamber phakic intraocular lenses for the correction of high myopia: anterior segment changes after posterior chamber phakic intraocular lens implantation (PMID: 11150270)	106	2001	Jiménez-Alfaro I
8	Toric Implantable Collamer Lens for moderate to high myopic astigmatism (PMID: 17198849)	101	2007	Sanders DR
9	Posterior chamber collagen copolymer phakic intraocular lenses to correct myopia: five-year follow-up (PMID: 21511154)	95	2011	Alfonso JF
10	Four-year follow-up of posterior chamber phakic intraocular lens implantation for moderate to high myopia (PMID: 19597102)	94	2009	Kamiya K

### 3.7 Co-occurrence analysis of keywords and citation bursts

**Figure 8:**
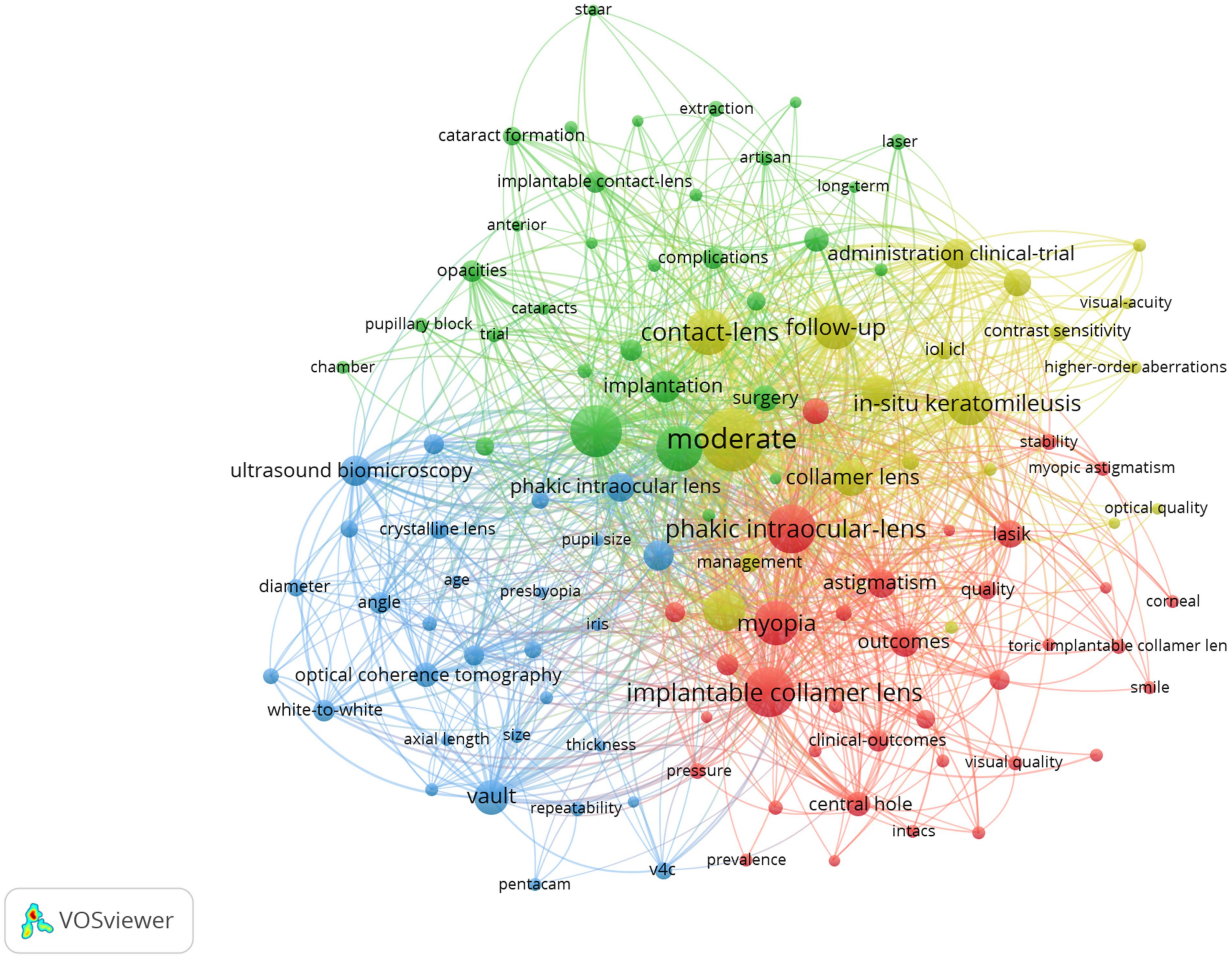
Co-occurrence network of keywords in posterior chamber pIOL research (The minimum number of occurrences of a keyword was set as 10; 117 of the 1,591 keywords involved in posterior chamber pIOL research met the threshold).

**Figure 9:**
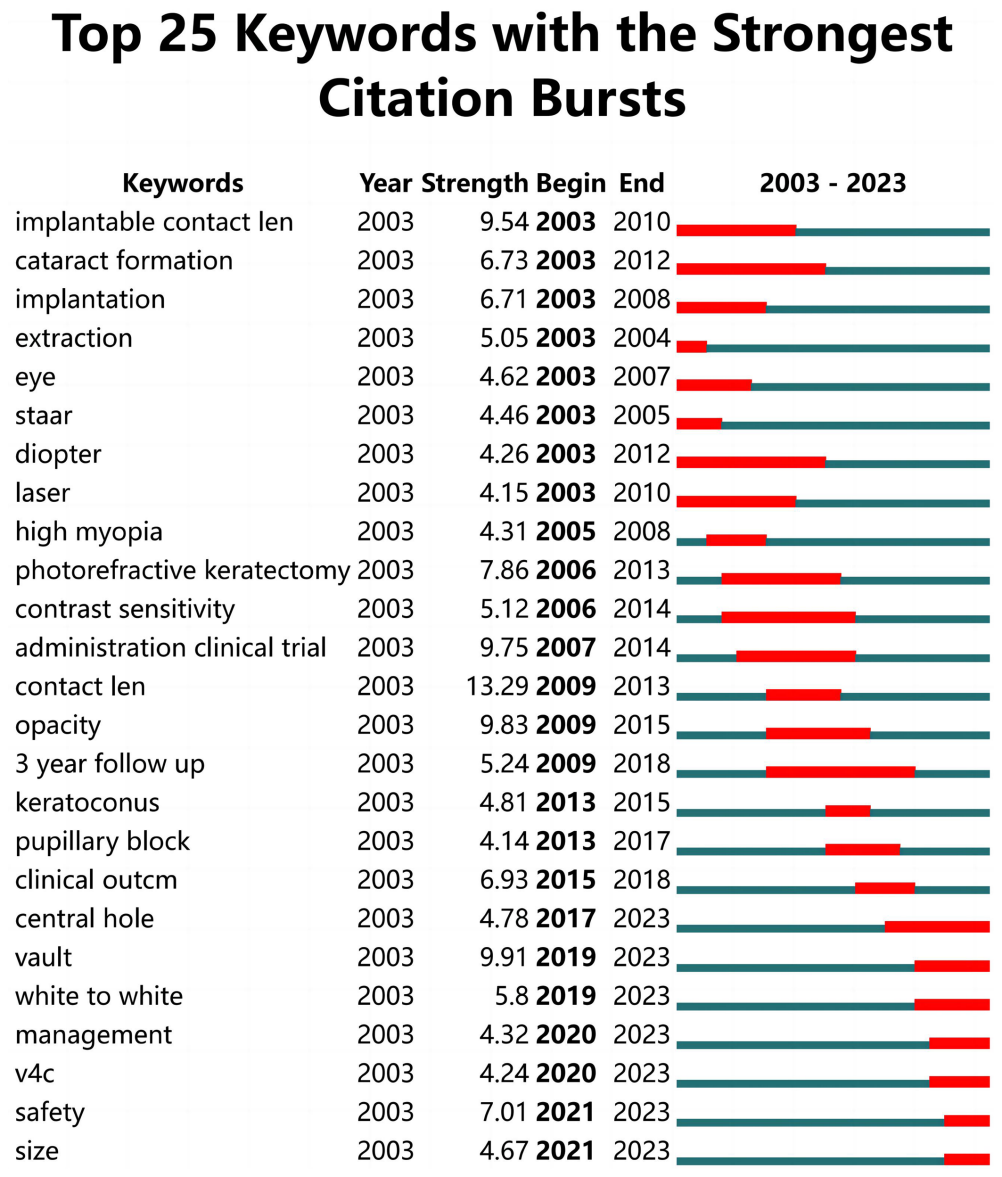
Top 25 keywords with the strongest citation bursts in posterior chamber pIOL research.

A high-frequency keyword co-occurrence analysis was conducted to identify the research topics in this field. A keyword co-occurrence network for studying posterior chamber pIOL was generated using VOSviewer. The minimum number of co-occurrences for a keyword was set at 10. Of the 1,591 extracted keywords associated with posterior chamber pIOL, 117 were grouped into four main clusters with red, green, blue, and yellow colors as indicators (Figure 8). Figure 9 shows the 25 keywords with the strongest citation bursts in this field between 2003 and 2023. After 2020, some of the popular keywords in the academic discourse were “management,” “v4c,” “safety,” and “size.”

## 4 Discussion

### 4.1 Global trends in research on posterior chamber pIOL

This study analyzed 791 original articles on posterior chamber pIOL published between 2003 and 2023. The findings indicate that there has been a consistent increase in the number of articles over the past two decades. This suggests that posterior chamber pIOLs are gaining acceptance and significant attention from the academic community. The number of publications in the past four years doubled, accounting for 43% of the total documents published in the last 20 years. This increase may be attributed to the bursts of keywords such as “management,” “v4c,” “safety,” and “size” in 2020. These findings indicate that the current research interests are centered on the perioperative management of posterior chamber pIOL and the safety and size selection of new generation v4c type ICL.

After publication location analysis, we discovered that research on this topic has been published in 62 countries and regions worldwide, indicating a global interest in posterior chamber pIOL. China, Spain, the United States, and Japan emerged as the top four contributors to productivity and influence among the 62 countries. In addition, our coauthor analysis revealed collaborations in this field across various countries, with the United States being central and exhibiting the highest total link strength. This suggests that the United States is a hub for international collaboration in posterior-chamber pIOLs.

It is possible to identify the most productive and influential organizations by examining the distribution of research institutions. Based on the findings presented in Table 2, the Kitasato University emerged as the top publisher and citation receiver, establishing itself as the most authoritative organization in this research field. The visualization diagram illustrates this further, with nodes representing the number of releases and links indicating collaboration. Figure 5 demonstrates that Fudan University (link = 9) and the Singapore National Eye Center (link = 9) have the highest number of connections, indicating strong collaborative ties with other institutions.

Building an author knowledge graph can provide valuable information to researchers seeking opportunities for collaboration. As shown in Table 3, Professor Shimizu Kimiya published 48 papers and was cited 1742 times, establishing him as a prominent figure in this research field. Figure 6 shows that the size of each node corresponds to the number of releases, and the strength of the links indicates the level of collaboration. We used coauthor analysis with the green and cyan groups to identify the four research groups that exhibited the highest global productivity in this field, indicating Prof. Xingtao Zhou (Fudan University, China) as the core.

The red group indicates Prof. Robert Montés-Micó (University of Valencia, Spain); the blue group indicates Prof. Kazutaka Kamiya (Kitasato University, Japan); and the yellow group indicates Prof. Takashi Kojima (Keio University, Japan) as their respective cores.

### 4.2 Intellectual base

We comprehensively elucidated the intellectual foundation and research context surrounding posterior chamber pIOL using co-citation analysis of publication references. Table 4 shows how the three co-cited references primarily examined the safety, effectiveness, and predictability of ICL surgery in addressing moderate to high myopic refractive errors and its impact on anterior subcapsular cataracts development, which ranked first in citation frequency and total link strength, indicating their central position in the knowledge network.

### 4.3 Research frontiers

Keyword co-occurrence analysis is a widely employed research method in bibliometrics that helps to uncover the primary internal knowledge structure and hotspot classification of relevant documents. Figure 8 illustrates that posterior chamber pIOL themes primarily form four clusters, with keywords in the same cluster sharing more similarities in research topics. Considering the characteristics and current state of posterior chamber pIOL research, we analyzed these four clusters.

Cluster#1 (red) focused on keywords associated with posterior chamber pIOL clinical outcomes for correcting myopic astigmatism. The frequently co-occurring keywords included myopia, outcomes, astigmatism, lasik, safety, central hole, keratoconus, stability, and penetrating keratoplasty. Studies have shown that ICL and toric ICL (TICL) are effective, safe, and predictable for myopia and myopic astigmatism correction (9–11). Multiple meta-analyses have demonstrated that ICL implantation can achieve comparable or superior effectiveness and safety in correcting moderate-to-high myopia compared with laser *in situ* keratomileusis (LASIK) and small incision lenticule extraction (SMILE) (5, 12–15). Notably, several clinical follow-up studies conducted over 5 years have consistently demonstrated the favorable stability of ICL and TICL (16–19). However, in super-high myopia cases with a diopter (D) < −12D, stability after ICL implantation may be slightly compromised, leading to continued myopia increase and axial growth (20, 21). Li et al. conducted a study in which ICL implantation was performed in the eyes of 60 patients with subclinical keratoconus. The findings revealed favorable postoperative efficacy, safety, and predictability, and the refractive outcomes remained stable throughout the 2-year follow-up period (22). Al-Amri et al. studied the clinical effects of TICL implantation in patients with stable keratoconus for >5 years (23). The results showed a significant improvement in the uncorrected visual acuity, changing from 20/248 preoperatively to 20/24 postoperatively. These findings suggest that TICL is a safe, effective, and stable treatment for vision enhancement. Alfonso-Bartolozzi et al. conducted a clinical observation of 15 eyes that underwent penetrating keratoplasty and received TICL for refractive error correction over 2 years (24). The results showed that 46.6 and 80% of the eyes achieved an uncorrected and corrected distance visual acuity (20/40), respectively. The safety index was 1.58, indicating TICL safety and effectiveness for residual myopia and astigmatism treatment after penetrating keratoplasty surgery. Currently, research on the use of posterior-chamber pIOL after corneal transplantation is limited. Further investigations are required to assess its predictability and safety.

Cluster #2 (green) focused on complications following posterior chamber IOL implantation. The frequently co-occurring keywords included high myopia, implantation, hyperopia, cataracts, complications, glaucoma, extraction, pupillary block, risk factors, and retinal detachment. Post-surgical cataract development is a frequent complication of posterior chamber pIOL. Cataract is primarily formed by placing the posterior chamber pIOL between the iris and lens, disrupting the circulation of aqueous humor around the lens. According to Vargas et al., cataract formation was the primary reason for bilensectomy following posterior chamber pIOL implantation, accounting for 93.1% of cases (25). Similarly, Hayakawa et al. discovered that the most prevalent cause of posterior chamber pIOL extraction is the progression of cataract formation, which accounted for 63% of cases (26). Meta-analyses show that cataract occurrence after ICL implantation was 1.1–5.9% before central-hole ICLs were introduced (27). Old age (> 40 years), high myopia (< −12.0D), and low vault (< 230 μm) are risk factors for cataract progression (27). In long-term follow-up studies of patients with central-hole ICLs over 5 years, the incidence of anterior subcapsular cataracts was 0.53%, whereas that of nuclear cataracts was 0.08% (28). Notably, nuclear cataract occurrence is associated with age and not influenced by ICL implantation (18). A 0.36-mm central hole facilitates the normal flow of aqueous humor, essential for maintaining proper fluid dynamics in the eye. This also enhances aqueous humor circulation around the lens, reducing anterior subcapsular cataracts (29).

Ocular hypertension is a common complication. Senthil et al. studied 638 eyes of 359 patients who underwent V4b and V4c model ICL implantation for 8 months. They found that 4.85% of patients developed intraocular pressure (IOP), whereas 0.3% developed glaucoma (30). The most common cause of increased IOP was steroid use, followed by viscoelastic agent residue and pupillary block (30). Another study by Naripthaphan et al. found no statistical difference in postoperative IOP between traditional ICL with peripheral iridotomy and central-hole ICL without a preoperative prophylactic incision (31). Qian et al. discovered that, in patients with shallow anterior chambers who underwent V4c model ICL implantation, a high vault may lead to narrowing of the anterior chamber, resulting in a long-term IOP increase. Therefore, in eyes with shallow anterior chambers, a narrower safe vault range is recommended (32).

Posterior chamber pIOL and other forms of inner eye surgery may pose a potential risk for vitreoretinal complications and retinal detachment (33). In their retrospective cohort study, Arrevola-Velasco et al. demonstrated that retinal detachment prevalence in patients who underwent ICL implantation over 10 years was 1.71% (34). The study found no evidence of increased retinal detachment risk in these patients compared with similar patients who did not undergo surgery (34). Myopia is a significant risk factor for rhegmatogenous retinal detachment, and its incidence increases with myopia severity (35). A strict fundus examination should be conducted before and after posterior chamber IOL implantation. In addition, preventive retinal laser photocoagulation can effectively mitigate the risk of retinal detachment if deemed necessary.

Cluster#3 (blue) focused on keywords associated with ICL size selection and postoperative vault predictions. Frequently co-occurring keywords included vault, ultrasound biomicroscopy (UBM), optical coherence tomography (OCT), angle, white-to-white, anterior segment, ciliary sulcus diameter, biometry, 3-year follow-up, v4c, size, pentacam, and anterior chamber depth. Currently, v4c ICL is the most widely used posterior chamber pIOL. The lens is available in four sizes, with lengths of 12.1 mm, 12.6 mm, 13.2 mm, and 13.7 mm (36). The postoperative vault, which is the distance between the posterior surface of the pIOL and the anterior surface of the crystalline lens, influences the risk of postoperative complications. Current methods for measuring vault height include UBM, anterior segment OCT, and Scheimpflug tomography (Pentacam). Studies have demonstrated that the vault height measurement value obtained from anterior segment OCT is higher than that obtained from UBM and Pentacam, with Pentacam showing the lowest measurement value (37). According to research, the ICL optimal vault typically falls within 250–750 μm (38–40). If the vault is >750 μm, it can result in the ICL pushing the iris forward, leading to changes in the angle shape, pupillary block, and an elevated risk of pigment dispersion glaucoma. Conversely, if the vault is too low (< 250 μm), cataract formation is more likely. ICL size selection is crucial in vault determination; therefore, optimizing the choice of ICL length is essential in reducing postoperative complications (41). According to the manufacturer’s recommended nomogram, ICL size selection has traditionally been based on anterior chamber depth and white-to-white diameter measurements (17). A meta-study conducted on 2,263 eyes across 24 studies revealed that considering a normal distribution of vaults, approximately 16% of eyes had vaults ranging from 0–250 μm, whereas 0.4% had vaults >1,000 μm (42). The inadequate vault can be partly attributed to the weak correlation between the white-to-white and ciliary sulcus diameters where the ICL was placed (43). Ciliary sulcus diameter measurement using UBM is a contact examination that requires the examiner to possess specific experience and is susceptible to subjective interference. This limits its widespread use in ICL size selection. A meta-analysis indicated that ICL-sizing methods based on sulcus-to-sulcus and white-to-white measurements do not yield clinically meaningful or statistically significant differences in the vault (42). Anterior segment OCT is a reliable and non-invasive method for obtaining anterior segment parameters. Research has shown that ICL size selection using anterior segment OCT multiple regression models or machine learning yields comparable or even superior outcomes compared with traditional nomograms (44–46). A crystalline lens rise was identified as an additional independent factor contributing to postoperative vault differences. It can be used for preoperative ICL-sizing calculations (47).

Cluster#4 (yellow) focused on keywords associated with postoperative visual quality following the posterior chamber pIOL implantation for myopia and astigmatism of varying diopters correction. The frequently co-occurring keywords included moderate *in situ* keratomileusis, follow-up, refractive surgery, administration clinical trials, photorefractive keratectomy, management, contrast sensitivity, higher-order aberrations, and spherical aberration. Compared with spectacle correction, ICL implantation has decreased intraocular scattering and enhanced optical quality in individuals with high myopia (48, 49). A study conducted on 42 patients who underwent ICL implantation for 1 year revealed a significant improvement in contrast sensitivity at 6, 12, and 18 cycles per degree after the procedure (50). Similarly, Bai et al. utilized the binoptometer 4P to measure contrast sensitivity and observed a significant enhancement compared with preoperative measurements (51). Shin et al. discovered that ICL implantation resulted in lower levels of ocular and corneal higher-order aberrations (HOA) in patients with highly myopic eyes than in those with wavefront-guided laser epithelial keratomileusis (52). Notably, multiple systematic reviews and meta-analyses have consistently demonstrated that high myopia ICL treatment yields a lower HOA than LASIK and SMILE (4, 5, 14). According to a study conducted by Tian et al., V4 ICL and central hole V4c ICL had comparable post-implantation visual qualities. However, V4c ICL resulted in higher levels of high-order and spherical aberrations (53). According to previous reports, some individuals experience visual interference known as “ring-shaped dysphotopsia” after ICL implantation. This phenomenon is associated with the refraction of stray light between the inner wall of the hole and the ICL posterior surface (54). However, it has been observed that patients can adapt to this interference within 6 months postoperatively (55).

### 4.4 Limitations

This study has some limitations. First, the analysis was based on publications between 2003 and 2023, which may not encompass all relevant topics in pIOL research. Second, the quality of published articles was not considered, and articles with varying research qualities were assigned equal weights. Finally, this study relied solely on data from the WoSCC database, potentially resulting in incomplete coverage of publications. Future studies should consider combining data from multiple databases to ensure a more comprehensive assessment.

## 5 Conclusion

This study presents the first bibliometric analysis of research trends in posterior chamber pIOL over the past two decades. We investigated the current state and emerging trends in global collaboration and research focal points in this field by visually analyzing pIOL-related research to offer researchers fresh insights and guidance.

## Author contributions

JN: Conceptualization, Methodology, Writing – original draft, Writing – review & editing. QZ: Conceptualization, Methodology, Writing – original draft, Writing – review & editing. WL: Data curation, Writing – original draft, Writing – review & editing. RZ: Visualization, Writing – original draft, Writing – review & editing. ZX: Software, Writing – original draft, Writing – review & editing. LJ: Validation, Writing – original draft, Writing – original draft. LZ: Writing – original draft, Writing – review & editing, Conceptualization.

## Footnotes

^1^ www.vosviewer.com
